# Supplementary material for: Dimensionality of the reinforced superconductivity in UTe2
Source: Nat Commun. 2025 Nov 21;16:10308. doi: 10.1038/s41467-025-66288-5 (PMC12638786; doi:10.1038/s41467-025-66288-5)
Supplement: Supplementary file 1 — Supplementary Information [file 41467_2025_66288_MOESM1_ESM.pdf]

Supplementary information for:

Dimensionality of the reinforced superconductivity in UTe_2

L. Zhang[1*], C. Guo[1*], D. Graf[2], C. Putzke[1], M. M. Bordelon[3], E. D. Bauer[3], S. M. Thomas[3], F. Ronning[3], P. F. S. Rosa[3], and P. J. W. Moll[1*]

[1]Max Planck Institute for the Structure and Dynamics of Matter, 22761 Hamburg, Germany

[2]National High Magnetic Field Laboratory, Tallahassee, FL 32310, USA

[3]Los Alamos National Laboratory, Los Alamos, NM 87545, USA

[*]Corresponding authors: ling.zhang@mpsd.mpg.de; chunyu.guo@mpsd.mpg.de; philip.moll@mpsd.mpg.de.

A. Sample information

The geometric dimensions of the three samples (S1, S2, S3) mentioned in the main text and the two samples (S4, S5) mentioned in this supplementary information are listed below in Supplementary Table 1. The SEM images of all samples are shown in Supplementary Fig. 1.

Sample No.	length (μm)	width (μm)	thickness (μm)*	current orientation
S1 (*J*//a)	22.64	2.544	4.459	*a*-axis
S1 (*J*//c)	10.69	3.307	4.459	*c*-axis
S2 (*J*//b)	16.19	3.309	5.456	*b*-axis
S2 (*J*//c)	10.40	2.825	5.456	*c*-axis
S3	9.459	3.109	4.257	*a*-axis
S4	48.72	6.106	4.853	*a*-axis
S5	3.208	1.986	1.356	*c*-axis

* thickness of each sample has a measurement error of ~100 nm, due to the uncertainty about the thickness of carbon capping layer on the top of the samples.

Supplementary Table 1. Geometries of all the measured samples

B. Strain effect of microstructure

The strain induced by mismatch of thermal expansion coefficients on our microstructure samples can be roughly estimated. The thermal strain of the sapphire substrate we used is about $\epsilon = -0.0008$ at 2 K, while the thermal strain of single crystal UTe_2 was measured in previous measurements[1], which gives $\epsilon_a = -0.00345, \epsilon_c = -0.00347$. Therefore, we can estimate that the thermal strain on microstructure when $T = 2$ K is about:

$$\epsilon = \epsilon^{UTe_2} - \epsilon^{sub} = -0.0027 \quad (1)$$

The Young's modulus of UTe_2 was also measured by other experiments[2], which is $E_a = 90.3$ GPa along a-axis and $E_c = 95.9$ GPa along c-axis. The stress on microstructure is:

$$\sigma_a = \epsilon \times E_a = -0.244 \text{ GPa} \quad (2)$$

$$\sigma_c = \epsilon \times E_c = -0.259 \text{ GPa} \quad (3)$$

The strain effect on T_c of UTe_2 was previously studied[3] and shows that for absolute stress in the range of 0~0.4 GPa, T_c shows a linear dependence on σ, with $\frac{dT_c}{d\sigma_a} = -0.87$ K/GPa, $\frac{dT_c}{d\sigma_c} = 0.56$ K/GPa. The change of T_c on our microstructure in different channels can then be estimated as:

$$\Delta T_c^{a-chn} = \sigma_a \frac{dT_c}{d\sigma_a} = 0.212 \text{ K} \quad (4)$$

$$\Delta T_c^{c-chn} = \sigma_c \frac{dT_c}{d\sigma_c} = -0.145 \text{ K} \quad (5)$$

$$\Delta T_c = \Delta T_c^{a-chn} - \Delta T_c^{c\ chn} = 0.357 \text{ K} \quad (6)$$

This estimation doesn't consider the distribution of stress on the whole sample, which may lead to an overestimation of the difference of T_c between two channels. In $\rho(T)$ curves, we observed the actual ΔT_c is about 0.15 K, which shows a good consistency with the above calculation.

C. Quantum oscillation

Here we compare the main frequencies in the Shubnikov-de Haas oscillations observed in our experiments when $H||c, J||a$ with previous reports on quantum oscillations of UTe_2 in Supplementary Table 2.

	This paper ($\theta = 91.3°$)	Broyles *et al.* 2023[4]	Eaton *et al.* 2024[5]	Aoki *et al.* 2023[6]
Type of experiment	4-point resistance	Tunnel diode oscillator	Magnetic torque and proximity diode oscillator	Field modulation
Frequencies	3.037 kT<br>3.3 kT<br>3.565 kT<br>3.864 kT	3.2 kT<br>3.7 kT<br>4.1 kT	3.5 kT	3.14 kT<br>3.33 kT<br>3.67 kT

Supplementary Table 2. Comparison of quantum oscillation frequencies when $H||c$ in different experiments

From the comparison, it is clear that the quantum oscillation frequencies measured in our transport measurement using FIB-fabricated microstructures are comparable to all other reports. However, our measurements present more details about the peak profile in the frequency range 3~4 kT, featuring four almost evenly spaced peaks with similar amplitudes. These four frequencies are closely located and can merge to a singular peak on the FFT spectrum dependent on angle, which may explain the singular frequency of 3.5 kT observed in previous torque and PDO measurement[5].

When the field is tilted away from *c*-axis, the peak profile of FFT spectrum in our measurements shows clear consistency with other reports. This indicates that the observation of these four oscillation frequencies may also require the precise alignment of the magnetic field with the crystallographic *c*-direction, which is easily achievable in microstructure samples.

D. Resistivity anisotropy calculation

To confirm that the resistivity anisotropy we observed at low temperature can be qualitatively explained by the quasi-2D Fermi surfaces of UTe_2, we calculated the conductivities σ_{ii} along three principal axes based on the Fermi surface proposed by Weinberger *et al.*[7], which is shown in Supplementary Fig. 2. Within relaxation time approximation, the electrical conductivity tensor is given by[8]:

$$\sigma_{ii} = \int \frac{d\mathbf{k}}{4\pi^3} \sum_n \tau_n(\mathbf{k}) v_{i,n}(\mathbf{k}) v_{i,n}(\mathbf{k}) \left(-\frac{\partial f}{\partial \varepsilon}\right)_{\varepsilon=\varepsilon_n(\mathbf{k})} \tag{7}$$

where n is the band index, and τ_n is the relaxation time for nth band. $v_{i,n}(\mathbf{k})$ is the ith component of the quasiparticle group velocity with momentum $\mathbf{k}$ in the nth band:

$$v_{i,n}(\mathbf{k}) = \frac{\partial \varepsilon_n(\mathbf{k})}{\hbar \partial k_i} \tag{8}$$

Using the $T = 0$ approximation, where $-\frac{\partial f}{\partial \varepsilon} = \delta(\varepsilon - \varepsilon_F)$, we obtain

$$\sigma_{ii} = \frac{1}{4\pi^3} \sum_n \oint \frac{\tau_n(\mathbf{k}_\mathrm{f}) v_{i,n}^2(\mathbf{k}_\mathrm{f})}{|v_{i,n}(\mathbf{k}_\mathrm{f})|} dS \tag{9}$$

Given the difficulty of accounting for electronic correlation, we only consider the geometric anisotropy encoded in the corrugated cylindrical Fermi surface, assuming a constant-valued Fermi velocity $v_{i,n}(\mathbf{k}_f)$. This means that, in this calculation, the Fermi velocity $v_{i,n}(\mathbf{k}_\mathrm{f})$ is replaced by the unit normal vector of the Fermi surface:

$$v_{i,n}(\mathbf{k}_\mathrm{f}) = n_{i,n}(\mathbf{k}_\mathrm{f}) \tag{10}$$

By further assuming an isotropic and constant relaxation time $\tau_n(\mathbf{k}_\mathrm{f})$, the calculated conductivity tensor reflects purely the anisotropy inherent in the geometry of a quasi-2D Fermi surface. The anisotropy of $\rho_c/\rho_{a,b}$ can then be expressed as:

$$\frac{\rho_c}{\rho_{a,b}} = \frac{\sigma_{a,b}}{\sigma_c} \tag{11}$$

With the above approximations, the calculation gives the following resistivity ratio:

$$\rho_c : \rho_a : \rho_b = 14.35 : 1.57 : 1 \tag{12}$$

This result exhibits anisotropy similar to that observed in our microbar measurement. First, the calculated in-plane anisotropy $\frac{\rho_a}{\rho_b} = 1.57$ agrees well with the experimental value $\frac{\rho_a}{\rho_b} \approx 2$. Given that we assumed only a simple form of scattering, this further supports our conclusion that the previously observed results[9] where $\rho_a < \rho_b$, are likely influenced by enhanced disorder

scattering, as evidenced by the significantly higher residual resistivity. Second, the calculated anisotropy $\frac{\rho_c}{\rho_b}$ is the highest, as expected for the cylindrical shape of the Fermi surface. Its value underestimates the experimental anisotropy by a factor of 3, which is not surprising given the purely geometric input of our electronic model in the absence of correlated behavior. Evidently, the anisotropy observed in our transport measurement is consistent with the quasi-2D Fermi surface picture supported by QO and ARPES experiments.

E. Phase boundaries compared to bulk crystals

In recent studies, differences among UTe_2 crystals grown by different methods have been discussed[10,11], in which the higher quality crystals with larger RRR have been confirmed to have higher T_c and broader superconducting regions of the SC1, SC2 phases in field and angle. In addition to T_c discussed in the main text, here we summarize the angular extents θ_{ba}, θ_{bc} of the SC2 phase (defined as the angle at which SC2 phase disappears) and compared with our results.

	Angle (degree)	Growth method	Ref.
θ_{ba}	4	CVT	Knebel *et al.* 2019[12]
	4.7	CVT	Ran *et al.* 2019[13]
	4	CVT	Lewin *et al.* 2024[14]
	7	MSF	Wu *et al.* 2024[11]
	6.4	MSF	Aoki *et al.* 2024[15]
θ_{bc}	12	CVT	Knebel *et al.* 2019[12]
	15	CVT	Lewin *et al.* 2024[14]
	20	MSF	Wu *et al.* 2024[11]
	23.8	MSF	Aoki *et al.* 2024[15]
	16	MSF	This work

Supplementary Table 3. Comparison of θ_{ba}, θ_{bc} in different transport studies.

Similarly, the critical field of metamagnetic transition H_m, also shows a certain amount of variation among all the previous experiments. Here we have collected reports on the value of H_m through resistivity measurements, in which we define H_m as the starting point of the transition where ρ deviates from zero. As can be seen, the value of H_m varies among crystals grown by different methods and is also dependent on current direction.

	Field (T)	Growth method	Ref.
H_m	33.14	CVT	Knafo *et al.* 2021[16]
	33.62	MSF	Aoki *et al.* 2024[15]
	33.74	MSF	This work (*J//c*)
	33.8	MSF	Knebel *et al.* 2024 (*J//a*)[17]
	33.8	CVT	Thebault *et al.* 2022 (*J//a*)[18]
	33.99	MSF	Wu *et al.* 2025[19]
	34.06	MSF	Wu *et al.* 2024[11]
	34.5	CVT	Knebel *et al.* 2024 (*J//b*)[17]
	34.59	CVT	Lewin *et al.* 2024[14]
	34.62	CVT	Ran *et al.* 2019[13]
	34.68	CVT	Knebel *et al.* 2024 (*J//c*)[17]

	34.79	MSF	This work (*J//a*)
	34.8	CVT	Thebault *et al.* 2022 (*J//c*)[18]

Supplementary Table 4. Comparison of H_m in different transport studies.

From the comparison, the phase boundaries of the SC2 and field polarized phase measured in our samples are within the variance from many previous transport studies on the same type of crystal, confirming the consistency of physical properties between FIB microstructures and bulk single crystals (Supplementary Table 3,4 and Supplementary Fig. 5).

F. Discussion of self-heating in microstructure

Here we carefully inspect the effect of self-heating in our FIB microstructures to exclude trivial origin of nonlinearity. We measure the IV characteristics of sample #5 at 44 T, 55.7 degrees, 0.3 K (Supplementary Fig. 6), which is in the paramagnetic normal state of the phase diagram and far away from either the SC phases or FP state. Here, it is a metal under the same thermal environment – providing an ideal situation to test self-heating effects via the increase of resistance under current bias.

At this field and angle the resistivity is further comparable to the flux-flow resistivity measured along c-axis in SC2 phase, therefore a similar degree of self-heating is expected. As shown in Supplementary Fig. 6, obvious deviation from linear behavior only occurs when the applied current density is higher than 15 kA/cm^2, which is about 50 times higher than the highest current density (J = 0.34 kA/cm^2), and more than 1000 times higher than the lowest current density (J = 0.0136 kA/cm^2) applied along c-axis channel in the dc+ac measurement in the main text.

G. Lorentzian fit of lock-in peak of flux-flow resistance

To extract more detailed information about the field-dependent behavior of the vortex lock-in peak, we conduct a peak fitting on groups of data points extracted from the field sweep data measured at several angles near 0 degrees. On each fixed-field slice, the data at different angles show clear enough peak features that justify a Lorentzian fit, for which the following Lorentz function is used:

$$\rho = \rho_0 + \frac{2A}{\pi}\frac{w}{4(x-x_c)^2+w^2} \qquad (13)$$

Where x_c is fixed to 0 as it was determined by careful rotations into the flux-flow maximum. The data points and the corresponding Lorentzian at 5 different field values are shown in Supplementary Fig. 7a. Despite only 5 data points for each fit, the standard error of the peak width w (Supplementary Fig. 7b) is small and the extracted field dependence $w(H)$ match well with the FWHM determined from the angle scans directly as shown in Fig. 3b.

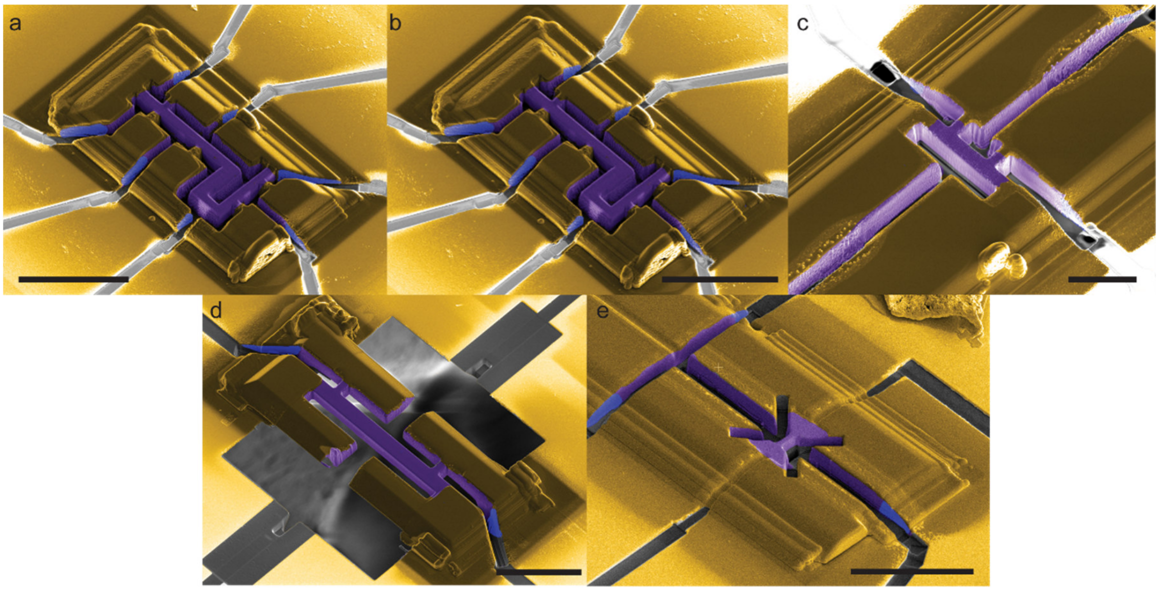

Supplementary Fig. 1. SEM images of all samples. The scalebars represent 20 μm. Parts of the samples colored yellow, purple, blue represent gold, UTe_2 and FIB-deposited platinum respectively.

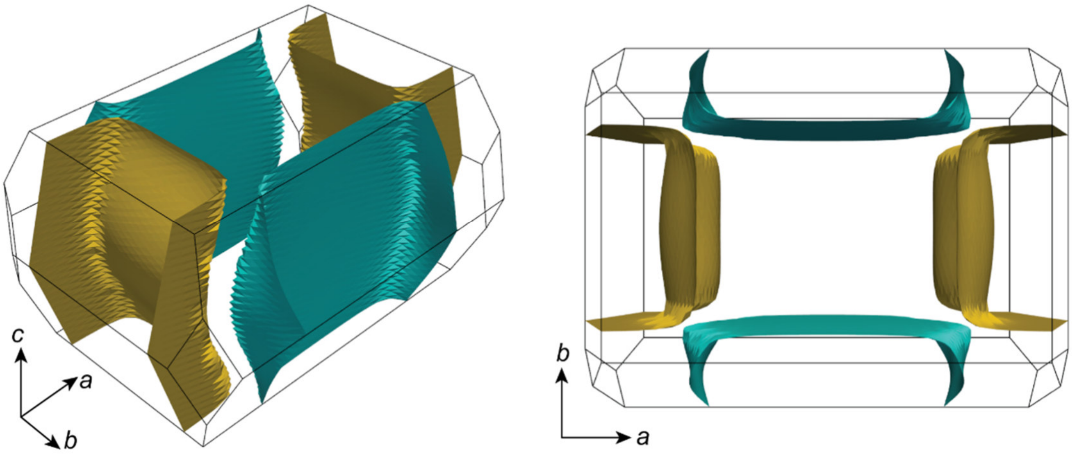

Supplementary Fig. 2. Calculated Fermi surface of UTe_2. The band structure is from the tight-binding calculation in Ref. 7.

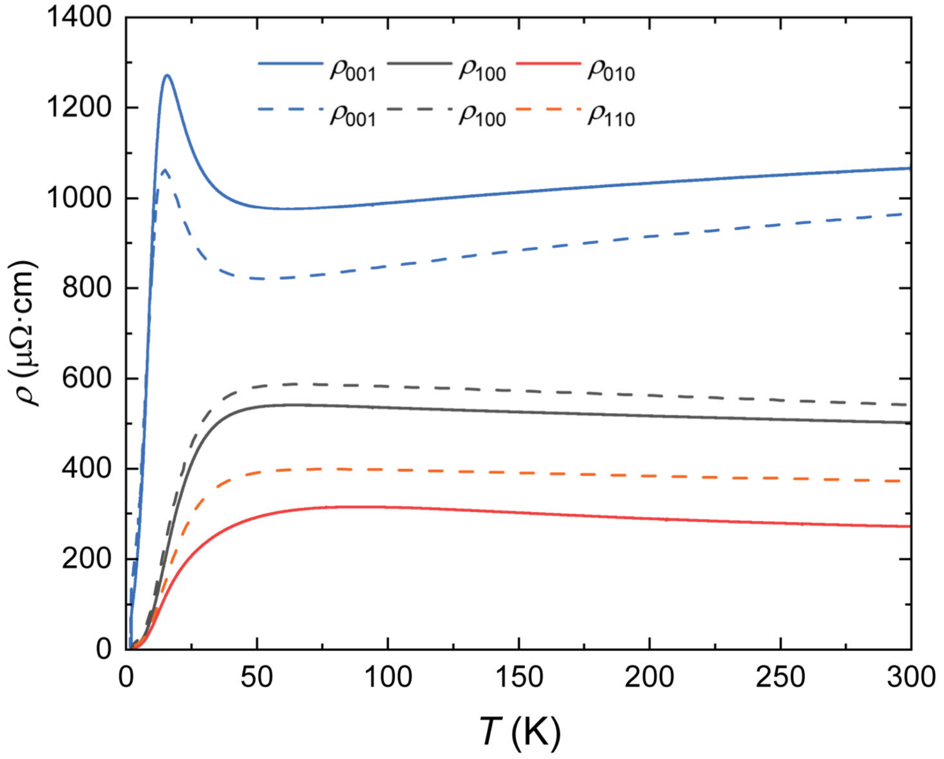

Supplementary Fig. 3. Comparison of resistivity of bulk and microbar samples. Resistivities measured from microbar and bulk single crystal are plotted with solid curves and dashed curves respectively. Data of bulk resistivity come from Ref. 3

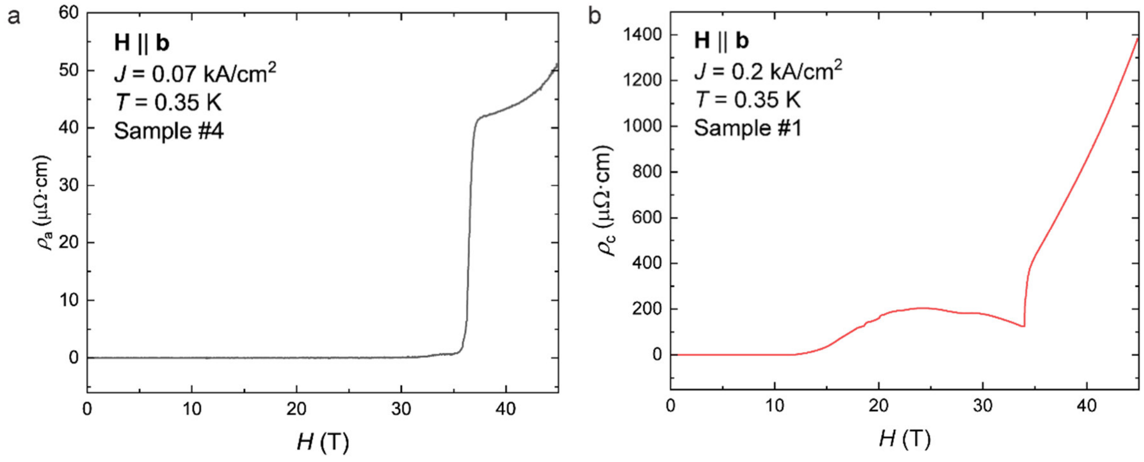

Supplementary Fig. 4. Magnetoresistivity with *b*-axis field down to 0T. a current along *a*-axis, **b** current along *c*-axis.

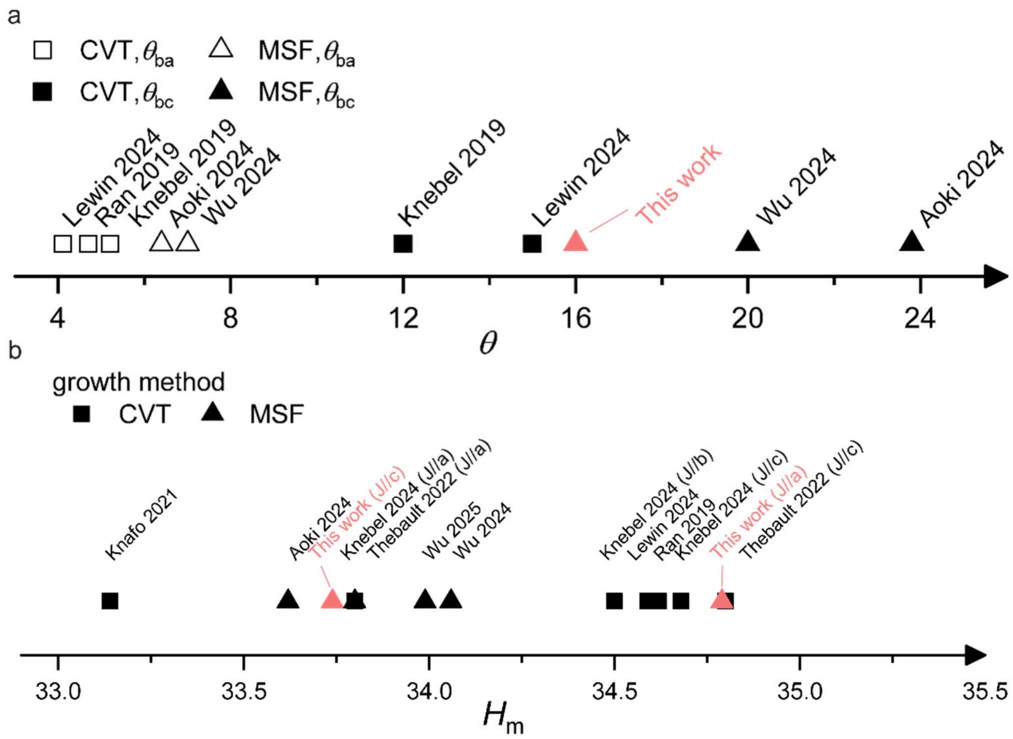

Supplementary Fig. 5. The variance of phase boundaries among different samples. **a** Angular ranges of SC2 phase from previous transport studies and this work. Filled and unfilled symbols represent measurements under *b*-to-*a* and *b*-to-*c* rotations, respectively. Rectangular and triangular symbols indicate data from CVT- and MSF-grown samples, respectively. **b** Critical field of the metamagnetic transition from previous transport studies and this work, with the same symbol conventions as in panel **a**.

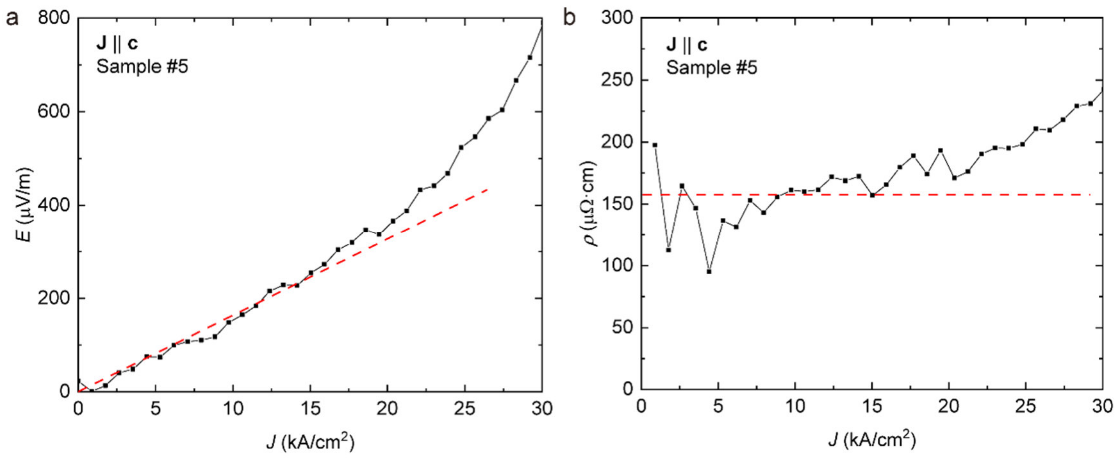

Supplementary Fig. 6. Check for self-heating. Nonlinear characteristics of sample S3 measured at $H = 44$ T, $\theta = 55.7°$, $T = 0.3$ K. **a** J-E curve, with dashed red line as a guide to the eye for linear (ohmic) behavior. **b** ρ-E curve, with dashed red line as a guide to the eye for constant resistivity.

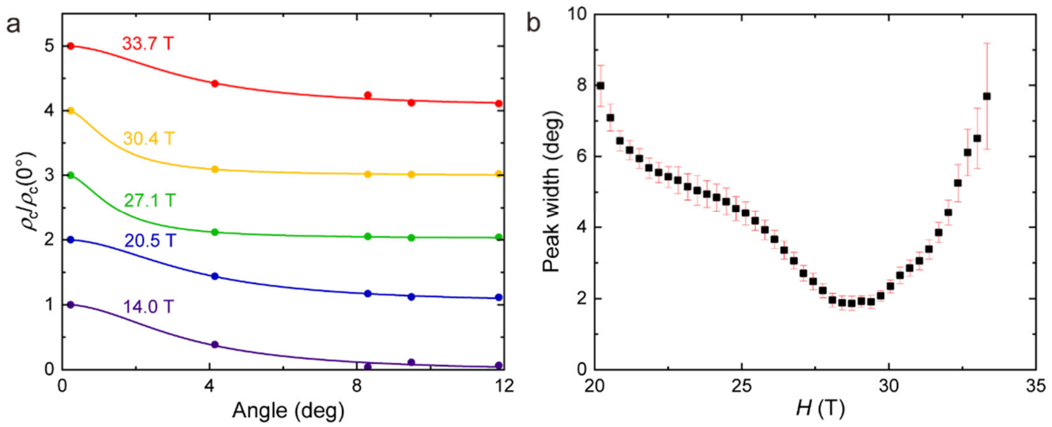

Supplementary Fig. 7. Lorentzian fit of field sweep data. a Lorentzian fit of data points at fixed field values from 5 field sweeps at different angles, offset by 1 for different fields. **b** The fitted peak width together with error bar showing the standard error of Lorentzian fit.

References

1. Thomas, S. M. *et al.* Spatially inhomogeneous superconductivity in UTe_2. *Phys. Rev. B* **104**, 224501 (2021).
2. Theuss, F. *et al.* Resonant Ultrasound Spectroscopy for Irregularly Shaped Samples and Its Application to Uranium Ditelluride. *Phys. Rev. Lett.* **132**, 066003 (2024).
3. Girod, C. *et al.* Thermodynamic and electrical transport properties of UTe_2 under uniaxial stress. *Phys. Rev. B* **106**, L121101 (2022).
4. Broyles, C. *et al.* Revealing a 3D Fermi Surface Pocket and Electron-Hole Tunneling in UTe_2 with Quantum Oscillations. *Phys. Rev. Lett.* **131**, 036501 (2023).
5. Eaton, A. G. *et al.* Quasi-2D Fermi surface in the anomalous superconductor UTe_2. *Nat. Commun.* **15**, 223 (2024).
6. Aoki, D. *et al.* De Haas–van Alphen Oscillations for the Field Along *c*-axis in UTe_2. *J. Phys. Soc. Jpn.* **92**, 065002 (2023).
7. Weinberger, T. I. *et al.* Pressure-enhanced f-electron orbital weighting in UTe_2 mapped by quantum interferometry. Preprint at https://doi.org/10.48550/arXiv.2403.03946 (2024).
8. Ashcroft, N. W. & Mermin, N. D. *Solid State Physics*. (Holt, Rinehart and Winston, New York, 1976).
9. Eo, Y. S. *et al.* *c*-axis transport in UTe_2: Evidence of three-dimensional conductivity component. *Phys. Rev. B* **106**, L060505 (2022).
10. Sakai, H. *et al.* Single crystal growth of superconducting UTe_2 by molten salt flux method. *Phys. Rev. Mater.* **6**, 073401 (2022).
11. Wu, Z. *et al.* Enhanced triplet superconductivity in next-generation ultraclean UTe_2. *Proc. Natl. Acad. Sci.* **121**, e2403067121 (2024).
12. Knebel, G. *et al.* Field-Reentrant Superconductivity Close to a Metamagnetic Transition in the Heavy-Fermion Superconductor UTe_2. *J. Phys. Soc. Jpn.* **88**, 063707 (2019).
13. Ran, S. *et al.* Extreme magnetic field-boosted superconductivity. *Nat. Phys.* **15**, 1250–1254 (2019).
14. Lewin, S. K. *et al.* Field-angle evolution of the superconducting and magnetic phases of UTe_2 around the b axis. *Phys. Rev. B* **110**, 184520 (2024).

15. Aoki, D. *et al.* High Field Superconducting Phases of Ultra Clean Single Crystal UTe_2. *J. Phys. Soc. Jpn.* **93**, 123702 (2024).
16. Knafo, W. *et al.* Comparison of two superconducting phases induced by a magnetic field in UTe_2. *Commun. Phys.* **4**, 40 (2021).
17. Knebel, G. *et al.* *c*-axis electrical transport at the metamagnetic transition in the heavy-fermion superconductor UTe_2 under pressure. *Phys. Rev. B* **109**, 155103 (2024).
18. Thebault, T. *et al.* Anisotropic signatures of electronic correlations in the electrical resistivity of UTe_2. *Phys. Rev. B* **106**, 144406 (2022).
19. Wu, Z. *et al.* A Quantum Critical Line Bounds the High Field Metamagnetic Transition Surface in UTe_2. *Phys. Rev. X* **15**, 021019 (2025).
